# A Case of Huntington's Chorea: Sir Clifford Allbutt's Clinical Lecture

**Published:** 1918-04-27

**Authors:** 


### A CASE OF HUNTINGTON'S CHOREA.

### Sir Clifford Allbutt's Clinical Lecture.

In the strict sense of the term a "clinical lecture," it will be accepted, is a lecture delivered at the bedside of a patient, and its text is the patient himself. The audience has something to see as well as something to hear, and the lecturer has to set an example of method and bearing, as well as to announce doctrines. The facts of the case necessarily take the first place, and the manner in which these are elicited is no small part of the teaching. There are fashions of observation and technical modes to be demonstrated, so that the hearers may learn the skill of craftsmanship. Not less important is the governing consideration that the inquiry has not only a scientific aim, but also the welfare of a human being as its object. Unfortunate indeed is the clinical enterprise which fails to remember this latter purpose. The audience is to be trained for the profession of medicine, and the human relation of doctor and patient must needs enter fully into the scheme. In short, the clinical lecture is not a set discourse, but an exhibition of the physician or surgeon engaged in his responsible and humane duty, and of this the care of the patient is the first law. As the facts accumulate they give rise to possible interpretations in the experienced mind of the teacher, and these—subject to the interest of the patient—are communicated to the audience, who thus follow the chain of reasoning which is prompted by the facts. If there are difficulties and doubts—and such are not infrequent in the study of clinical problems—these are frankly stated, and fact and opinion have each its appropriate place. In short, the clinical lecture is a clinical study of the individual patient, his symptoms, the meaning of his symptoms, and his needs, and in this study both the teacher and his students have an active part to play. The question is not—What can be told about a given disease? Rather it is—What are the facts in this individual patient, and how are they related one to the other? The undertaking is a study in natural history touched in greater or less measure by the pathos of human suffering. It is, or ought to be, an education in the medical art in the fullest sense of this term, and a training in method and manner and fact which will remain with the student as an abiding personal possession. The generalisations of the text-book may stay the demands of an examination, but the clinical exercises of a great teacher are the true training-ground for practice and for life.

From what is here said it must be obvious that to produce the full value of the clinical lecture on the printed page is an almost impossible task. There are delicacies of fashion and statement which necessarily escape reproduction, and which are no small part of the discipline enjoyed by the audience. But for our own part we could wish that some of our great clinical teachers would more often make the experiment of publishing their bedside studies. Here is the very truth of medicine as Nature actually presents it, the difficulties and limitations which meet the practitioner in his daily work. No teaching is more valuable and so much to the purpose. It may perhaps be said that clinical lectures are a fairly prominent feature in current professional literature, and the term, be it allowed, is not an infrequent one. But between the term and the thing there is often, and even commonly, a wide gap. Not every printed article which invitingly announces itself as a "clinical lecture" merits this dignity, at least as we understand the phrase. The experience is a familiar one to find a lecture published as "clinical" which from start to finish contains no reference to an individual patient and which, still less, has such a patient for its text. The display of learning may be magnificent, but it is not—clinical. At least such lectures as we have in mind cannot be distinguished from a systematic series in which a topic is treated on the basis of a wide review of existing knowledge. Far be it from us to say that such discourses are without value. On the contrary, we recognise that they have their place and justification in the educational scheme, and we expect to find them in every well-organised text-book. But why they should be called "clinical" we have never been able to discover, for they are only related to the facts and difficulties of clinical medicine in the same way as the doctrines of chemistry are related to the cause and consequence of an individual explosion. Of lectures of this order we have enough and to spare. But the genuine clinical study in which we get a clean-cut picture of an individual patient might with advantage well be multiplied. The laboratory nowadays has much to say, and its teaching is welcome and helpful. But the patient's bedside is the real training-ground for the practitioner, and to learn how the masters of the art there comport themselves has the influence of a liberal education.

These remarks have been prompted by the appearance in the *British Medical Journal* of a clinical lecture on Huntington's chorea by Sir Clifford Allbutt; and it is the reading of this lecture which has led us to express the desire for more expositions of the same order. Cases of Huntington's chorea are admittedly rare experiences; but they occur, and therefore there is need to recognise them. What follows from failure in this direction is illustrated in the present instance, as the unfortunate sufferer had been sent from hospital to hospital with the unsympathetic label of "neurasthenia." His jerks and shufflings and contortions under such a label could hardly expect much forbearance from a committee of persons who know by experience that "neurasthenia" is, at least sometimes, a name for a condition of more perverse character. Fortunately, on this occasion, Sir Clifford Allbutt was a member of the committee, and was able both to help his colleagues to a right conclusion and to secure the patient for the benefit of his clinical

**HUNTINGTON'S CHOREA.**—(*continued*).

class. The fortunate members of this class have the first advantage we doubt not; but the readers of the lecture are not far behind. There is the picture drawn from life by the pencil of a great clinical artist. Those who will may view it, and we venture to say that those who do so will have the sense of a personal experience. They have not actually seen the case, it is true. But they may read of the manner in which better eyes than their own have seen it, and this may perchance be more impressive than a direct but uninstructed vision.

